# Gastric peroral endoscopic myotomy for compassionate treatment of symptomatic gastroparesis in patients on chronic opioids

**DOI:** 10.1016/j.igie.2025.05.001

**Published:** 2025-05-30

**Authors:** Maham Hayat, Abel Joseph, Yasi Xiao, Abdullah Abbasi, Saurabh Chandan, Sagar Pathak, Baha Aldeen Bani Fawwaz, Aimen Farooq, Natalie Cosgrove, Deepanshu Jain, Mustafa A. Arain, Kambiz Kadkhodayan, Ernesto R. Gonzaga, Irteza Inayat, Muhammad K. Hasan, Joo Ha Hwang, Dennis Yang

**Affiliations:** 1 Center for Interventional Endoscopy, AdventHealth, Orlando, Florida, USA; 2 Division of Gastroenterology and Hepatology, Stanford University School of Medicine, Palo Alto, California, USA; 3 Gastroenterology and Hepatology, AdventHealth Medical Group, Orlando, Florida, USA

## Abstract

**Background and Aims:**

It has been estimated that nearly half of patients with gastroparesis use opioids long term, are unable to cease or wean opioid use, and continue to endorse debilitating symptoms. The aim of this study was to evaluate outcomes of gastric peroral endoscopic myotomy (G-POEM) as compassionate treatment in patients with gastroparesis who are unable to discontinue long-term opioid use.

**Methods:**

This was a multicenter retrospective analysis of prospective databases containing patients with gastroparesis taking opioids who underwent G-POEM between January 2021 and November 2024. Functional luminal imaging probe (FLIP) pyloric measurements were obtained pre- and post-G-POEM at follow-up. Clinical response to G-POEM was defined as improvement of ≥1 point on the Gastroparesis Cardinal Symptom Index.

**Results:**

Thirty-eight patients with gastroparesis on opioids with a median follow-up of 6 months (range, 3-18 months) underwent G-POEM during the study period. Technical success was 100%. Clinical success was achieved in 60.5% (23/38) of the patients. There were no differences in demographics, symptom duration, etiology of gastroparesis, baseline Gastroparesis Cardinal Symptom Index score, gastric-emptying scintigraphy or daily morphine milligram equivalent between responders and nonresponders. When compared with nonresponders, patients who responded to G-POEM had a significantly lower FLIP baseline pyloric diameter (13.2 [standard deviation (SD) 3.4 mm^2^ ] vs 15.6 [SD 2.7 mm^2^]; *P* = .04) and distensibility index (4.8 [SD 1.9 mm^2^/mm Hg] vs 8.4 [SD 4.6 mm^2^/mm Hg]; *P* = .004). Clinical response was associated with improvement in FLIP pyloric diameter and distensibility index.

**Conclusions:**

This study suggests that G-POEM could be considered as a safe and effective compassionate treatment in select patients with gastroparesis who are taking opioids long term, particularly those with lower pyloric compliance on physiologic testing. Future studies are needed to corroborate these preliminary findings.

Gastroparesis is a syndrome characterized by symptomatic delayed gastric emptying in the absence of mechanical obstruction. Patients commonly experience disabling symptoms, including nausea, vomiting, early satiety, bloating, abdominal pain, and weight loss, which often translates into frequent hospital visits and a financial burden to the health care system.^1^^,^^2^

The underlying causes of gastroparesis are diverse, including diabetes, postsurgical, and in many instances idiopathic.^1^ Medications that delay gastric emptying can either cause or worsen symptoms in patients with gastroparesis. Among these, the use of opioids in gastroparesis has been increasing, with some studies reporting that 30% to 46% of patients with gastroparesis are taking opioids long term.^3^, ^4^, ^5^ Management should revolve around discontinuation of opioids and use of nonopioid medications and nonpharmacologic therapies for pain. Nonetheless, this approach is not realistically feasible in most patients taking opioids long term.^6^

With limited options for patients with medically refractory gastroparesis, there has been a resurgent interest in pyloric-directed therapies with the advent of gastric peroral endoscopic myotomy (G-POEM).^7^^,^^8^ Although studies have reported clinical response to G-POEM ranging from 50% to 80%, there is a scarcity of data focusing on the role of this procedure in the setting of opioid use. In this multicenter study, we aimed to evaluate clinical outcomes of G-POEM as a compassionate treatment for symptomatic gastroparesis in patients unable to discontinue long-term opioid use.

## Methods

### Study design and population

This was a 2-center retrospective analysis of prospectively collected data of consecutive patients ≥18 years of age who underwent G-POEM between January 2021 and November 2024. Patients were included in the study if they met the following inclusion criteria: (1) gastroparesis refractory to standard treatment (including dietary modification and prokinetics), (2) delayed gastric emptying >10% meal retention at 4-hour solid-phase gastric-emptying scan (GES),^7^ and (3) opioid use as confirmed in the patient's medication list. Long-term opioid use was defined as daily opioid use at the time of G-POEM. Before G-POEM eligibility, all patients were asked whether an attempt was made to either discontinue or at least reduce opioid use as part of their management of gastroparesis. Only patients who had a previous attempt to discontinue/wean opioids yet with persistent symptoms of gastroparesis were included in the study. Patients were excluded if they met any of the following criteria: (1) upper gastrointestinal (GI) surgeries involving the pylorus, including previous surgical or laparoscopic pyloromyotomy, (2) current or history of gastroesophageal malignancy, (3) pregnancy, (4) any contraindication to endoscopy, and/or (5) those unable to provide informed consent. The study was approved by the institutional review board for human research at each participating institution, with the Center for Interventional Endoscopy at AdventHealth, Orlando, Florida, United States, serving as the central coordinating center. All authors had access to the study data and reviewed and approved the final manuscript.

The primary aim of the study was to evaluate clinical response to G-POEM among patients on opioids. There are currently no data on G-POEM outcomes in this study population. However, previous studies have suggested that pain tends to be a negative predictor of response.^1^ On the basis of this information, our hypothesis was that patients on opioids would experience a lower clinical response rate with G-POEM for the management of symptomatic gastroparesis.

### G-POEM procedure

All patients underwent endoscopy under propofol sedation and carbon dioxide insufflation. The G-POEM procedures were performed by providers (D.Y., M.K.H., J.H.H.) with experience in submucosal endoscopy. The procedure was performed as previously described in the literature.^1^^,^^8^^,^^9^ In brief, all cases were performed with the patient in left lateral decubitus position. Patients received 3.75 g of piperacillin/tazobactam or 500 mg of ciprofloxacin shortly before the procedure. A standard gastroscope (GIF-H190; Olympus, Tokyo, Japan) with a clear distal attachment cap (MH-588; Olympus America, Center Valley, Pa, USA) was used for all procedures.

A submucosal injection of 6% hetastarch admixed with methylene blue was performed approximately 5 cm proximal from the pylorus on the greater curvature. After the submucosal lift, a 1.5- to 2.5-cm mucosal incision was made (ENDO CUT Q, effect 3, cut duration 2, cut interval 1) (Erbe, Marietta, Ga, USA) with the electrosurgical knife (Hybrid I-type knife, Erbe; TT-J knife, Olympus America). Submucosal dissection was then performed with the electrosurgical knife (ENDO CUT Q and forced coagulation, effect 2, maximum 50 W) and repeated submucosal injections until the pyloric ring was exposed. Pyloromyotomy was then performed by starting at the pylorus ring from the most distal aspect of the pylorus to proximal and extended approximately 2 cm toward the antrum. Finally, the mucosal incision was closed with endoscopic clipping and/or suturing.^8^^,^^10^

### Functional luminal imaging probe

**Figure 1:**
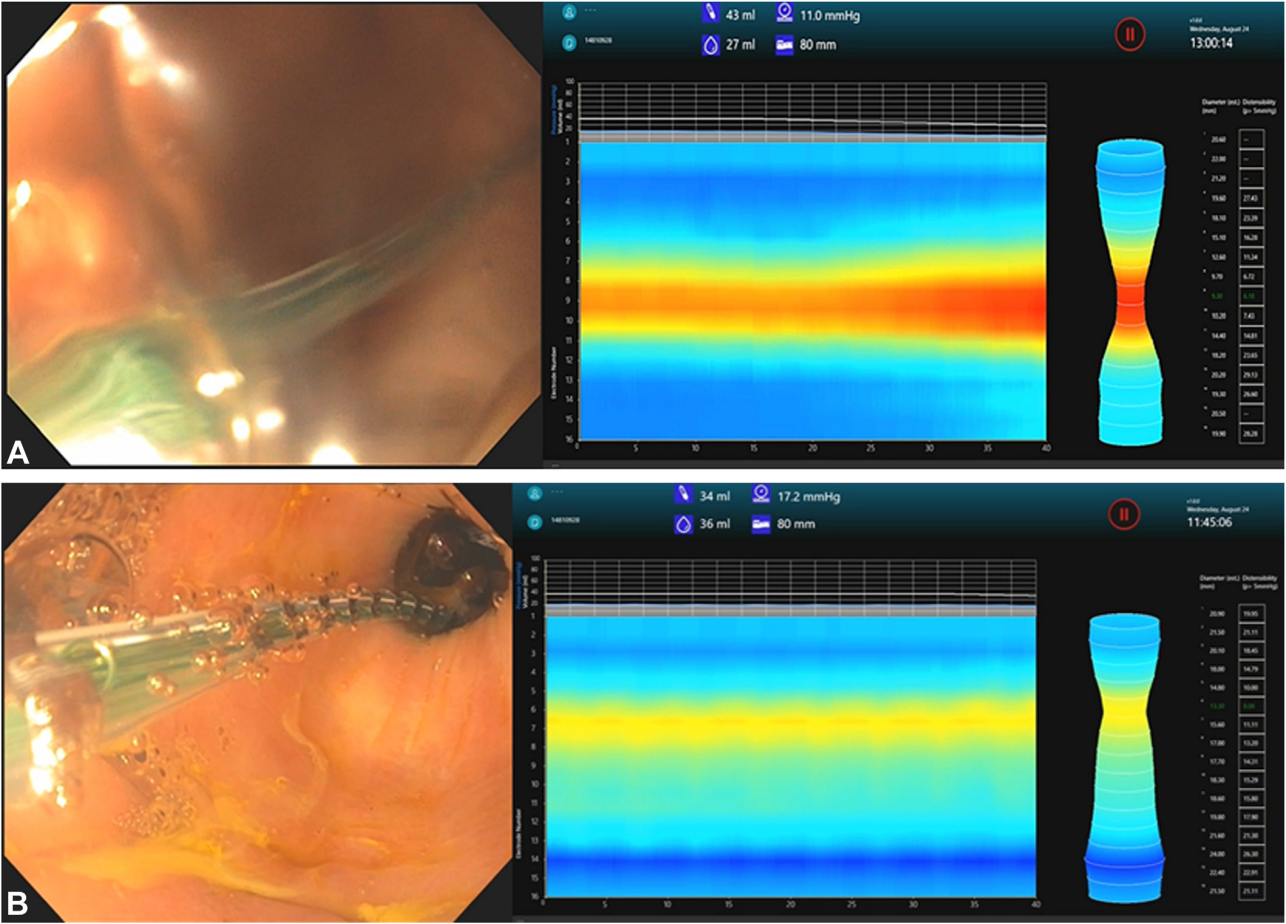
Functional luminal imaging probe pyloric measurements pregastric peroral endoscopic myotomy (G-POEM) **(A)** and post-G-POEM **(B)** in a patient taking opioids long term, with symptomatic improvement after intervention. The pyloric diameter and distensibility index were 9.3 mm and 6.18 mm^2^/mm Hg pre-G-POEM and 13.3 mm and 8.08 mm^2^/mm Hg post-G-POEM, respectively.

Intraluminal measurements of the physiologic characteristics of the pylorus were obtained using the commercially available functional luminal imaging probe (FLIP) technology (models EF-100 and EndoFLIP 2.0, balloon catheter EF-325N; Medtronic, Minneapolis, Minn, USA) (Fig. 1). At the Center for Interventional Endoscopy, a therapeutic gastroscope with a 6.0-mm accessory channel (GIF-XTQ160; Olympus) was used. With the gastroscope in the second portion of the duodenum, the FLIP catheter was carefully advanced through the channel with simultaneous withdrawal of the scope into the stomach until the FLIP catheter was positioned across the pylorus. At the other participating center, the FLIP catheter was advanced adjacent to the upper endoscope (GIF-HQ190; Olympus) by holding the tip of the FLIP catheter with a snare. Once in the second portion of the duodenum, the snare was opened to release the FLIP, and this was slowly pushed in while withdrawing the endoscope. Finally, with the endoscope in the stomach, most of the FLIP catheter was positioned in the antrum as to minimize deformation of the balloon.^11^ The FLIP balloon was stabilized and inflated to a fixed volume of 40 mL in all cases. The following measurements were obtained after waiting at a fixed volume for approximately 30 seconds to minimize variations during peristaltic contractions: intraballoon pressure, pylorus diameter, cross-sectional area, and distensibility index (DI).^12^^,^^13^

### Data collection

Relevant data were abstracted from patient chart, endoscopy reports, and prospectively maintained registries. Data on patient demographics, gastroparesis (etiology, duration of symptoms, Gastroparesis Cardinal Symptom Index [GCSI] score, previous interventions), GES, medications, were collected. Procedure data included pyloric FLIP measurements, length of pyloromyotomy, procedure time, technical success of G-POEM, and adverse events. Postprocedure data (ie, GCSI, pyloric FLIP) also were recorded at the time of follow-up. There was no study-specific follow-up protocol. Patients were followed as per standard of care at each participating institution, which included scheduled postoperative clinic visit (usually within 3-6 months from the procedure) and/or repeat endoscopy with FLIP within the same timeframe.

### Definitions and study end points

Total opioid use was expressed in morphine milligram equivalents (MMEs). Technical success was defined as successful completion of the G-POEM procedure as intended. Clinical response was assessed using the GCSI score. The GCSI includes 3 subscales: postprandial fullness/early satiety, nausea/vomiting, and bloating. In concordance with G-POEM literature, clinical response was defined as an improvement of ≥1 point on GCSI at the time of follow-up.^14^ Adverse events were graded according to a standardized adverse events classification system for GI endoscopy.^15^

### Statistical analysis

All variable and outcome distributions were summarized as percentages for categorical variables and means with standard deviation and median with interquartile range (IQR) for all continuous variables. The Shapiro-Wilk test was used to determine normality. If variables were determined to follow a normal distribution, they were compared using an independent sample *t* test. Nonparametric testing was performed on those that did not assume a normal distribution, including the 2-sample Wilcoxon rank-sum test. Dichotomous variables were evaluated in a univariate fashion using a Pearson χ^2^ test. If the sample size for a dichotomous variable was not sufficiently large enough, the Fisher exact test was used. A *P* value <.05 was used to determine significance. All analyses were performed using SAS, version 9.4 (SAS Institute, Cary, NC, USA).

## Results

### Study population

Thirty-eight patients (median age, 53 years; IQR, 35-58 years; 78.9% women) with symptomatic gastroparesis on chronic opioids underwent G-POEM between January 2021 and September 2024. In addition to opioid use, contributory etiologies for gastroparesis included diabetes (n = 15), postsurgical (n = 9), and idiopathic (n = 14). Twenty-one of the 38 patients (55.3%) had concomitant psychiatric comorbidities (anxiety, n = 20; depression, n = 17). Most patients (n = 32; 84.2%) had previous botulinum toxin injections to the pylorus. Baseline mean 4-hour retention percentage on GES and baseline GCSI were 49.3 (standard deviation [SD] 19.8) and 7.1 (SD 4.21), respectively. The median daily MME was 41 (IQR: 29-58). The 3 most common opioids taken by patients were morphine (n = 11), oxycodone (n = 10), and hydrocodone (n = 8).

### Clinical response to G-POEM

**Figure 2:**
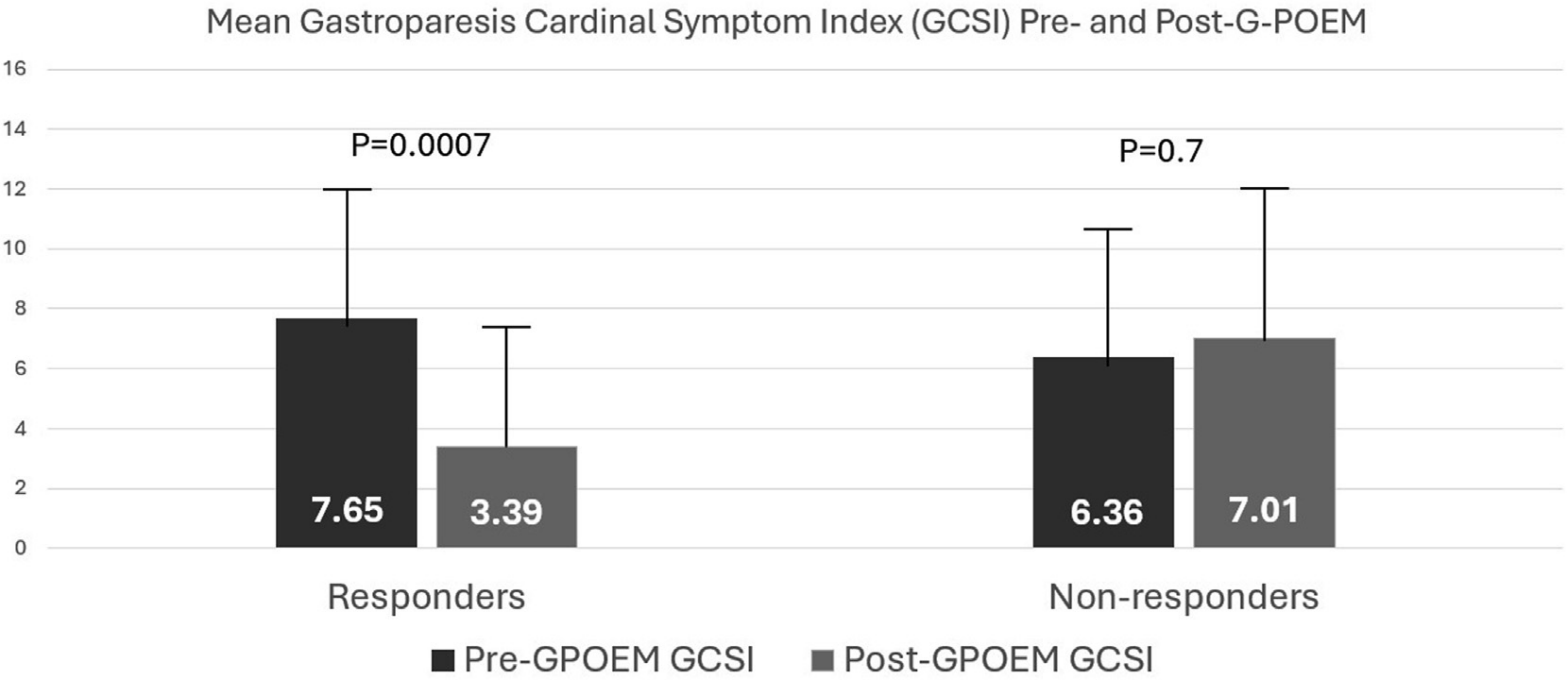
Mean Gastroparesis Cardinal Symptom Index (GCSI) scores pre- and postgastric peroral endoscopic myotomy (G-POEM).

Twenty-three of the 38 patients (60.5%) reported sustained clinical response at a median follow-up of 6 months (IQR: 3-12 months). Figure 2 compares the pre- and post-G-POEM GCSI scores between responders and nonresponders. When compared with nonresponders, there was a significant decrease in the mean GSCI score among responders after G-POEM (7.65 ± [SD 4.08] vs 3.39 [3.86 SD], *P* = .0007).

Table 1 summarizes characteristics between G-POEM responders versus nonresponders. There were no differences in patient demographics, duration of gastroparetic symptoms before G-POEM, baseline GES, or mean GCSI scores between the 2 groups (Table 1). Likewise, in terms of opioid use, there was no difference in the type of opioids or total daily MME between clinical responders versus nonresponders. Procedural characteristics (ie, length of pyloromyotomy, procedure time) were similar between responders versus nonresponders. There was 1 case (2.6%) of delayed bleeding. This patient presented on postoperative day #2 with melena. Radiographic imaging and endoscopy did not reveal active bleeding, and the patient did not require blood transfusions. She was discharged after 1 day of hospital observation with no additional issues at 4-week follow-up.

**Table 1:** Baseline and procedural characteristics

Baseline characteristics	Responders (n = 23)	Nonresponders (n = 15)	*P* value
Age, years, median (IQR)	49 (38-58)	51 (35-56)	.82
Female sex, n (%)	18 (78.3)	12 (80)	1.00
Duration of symptoms, mo, median (IQR)	36 (24-60)	48 (33-12)	.67
Etiology of gastroparesis, n (%)			
Diabetes mellitus	8 (34.8)	7 (46.7)	.51
Postsurgical	6 (26.1)	3 (20)	1.00
Idiopathic	9 (39.1)	5 (33.3)	1.00
Comorbidities, n (%)			
Anxiety	12 (52.2)	8 (53.3)	1.00
Depression	13 (57.9)	4 (26.7)	.10
Previous botulinum toxin injection to pylorus, n (%)	20 (86.9)	12 (80)	1.00
Daily MME, median (IQR)	42.5 (29-60)	40 (30-45)	.88
Type of opioid, n (%)			
Oxycodone	6 (26.1)	4 (26.7)	1.00
Tramadol	1 (4.3)	1 (6.7)	1.00
Morphine	6 (26.1)	5 (33.3)	.72
Hydromorphone	5 (21.7)	2 (13.3)	.68
Hydrocodone	5 (21.7)	3 (20)	1.00
Buprenorphine	2 (8.7)	1 (6.7)	1.00
Roxicodone	2 (8.7)	0	.51
Indication for opioid use, n (%)			
Back pain	13 (52.2)	8 (53.3)	1.00
Pelvic pain	4 (13.0)	2 (13.3)	1.00
Abdominal pain	5 (21.7)	5 (33.3)	.47
Endometriosis	1 (4.3)	0	1.00
Meal percentage retention at 4-hour on gastric-emptying scan (GES); median (IQR)	62 (51-84)	56 (39-78)	.78
Baseline gastroparesis Cardinal Index Score, mean (SD)			
Nausea/retching/vomiting	3.46 (0.97)	3.00 (1.11)	.22
Stomach fullness/loss of appetite	4.27 (0.58)	4.17 (0.96)	.72
Bloating/distension	3.27 (1.71)	3.89 (1.54)	.30
Global	7.65 (4.08)	6.36 (4.31)	.40
Procedural characteristics			
Length of pyloromyotomy, median (IQR)	2.0 (2-2)	2 (2-2.25)	1.00
Total procedure time, median (IQR)	41 (38-47)	42 (41-50)	.72
Adverse events, n (%)			
Delayed bleeding	1 (4.3)	0	1.00
Perforation	0	0	1.00

*IQR*, interquartile range; *MME*, morphine milligram equivalent; *SD*, standard deviation.

### Pyloric FLIP, MME intake, and response to G-POEM

Pyloric FLIP measurements (pre-G-POEM and at follow-up) were available for all 38 patients. The baseline and post-G-POEM FLIP measurements for both responders and nonresponders are summarized in Table 2. Compared with nonresponders, patients who responded to G-POEM had a significantly lower mean baseline pyloric diameter (13.20 [SD 3.43 mm^2^ ] vs 15.6 [SD 2.7 mm^2^ ]; P = .04) and DI (4.8 [SD 1.9 mm^2^/mm Hg] vs 8.4 [SD 4.6 mm^2^/mm Hg; *P* = .004). Notably, following G-POEM, there was a significant increase in both pyloric diameter and DI in the clinical responder group but not in the nonresponder group (Table 2).

**Table 2:** Pre- and post-G-POEM FLIP pyloric measurements for clinical responders and nonresponders

	Pre-G-POEM	Post-G-POEM	*P* value
Clinical responders (n = 23)			
Diameter, mm, mean (SD)	13.2 (3.4)	17.2 (3.0)	.0001
Cross-sectional area, mm^2^, mean (SD)	161.8 (63.7)	236.4 (75.8)	.0008
Pressure, mm Hg, mean (SD)	30.4 (11.3)	28.2 (14.4)	.57
Distensibility Index (DI), mm^2^/mm Hg, mean (SD)	4.8 (1.9)	10.7 (5.2)	<.0001
Clinical nonresponders (n = 15)			
Diameter, mm, mean (SD)	15.6 (2.7)	15.1 (2.4)	.59
Cross-sectional area, mm^2^, mean (SD)	208.1 (86.3)	180.7 (49.9)	.30
Pressure, mm Hg, mean (SD)	24.5 (10.5)	31.0 (9.2)	.08
Distensibility Index (DI), mm^2^/mm Hg, mean (SD)	8.4 (4.6)	7.6 (1.9)	.54

*FLIP*, Functional luminal imaging probe; *G-POEM*, gastric peroral endoscopic myotomy; *SD*, standard deviation.

The MME intake after G-POEM was assessed. There was a decrease in MME intake in 2 of 23 patients (8.7%) in the responder group, whereas there was no change (0%, 0/15) in MME intake in any of the patients in the nonresponder group (*P* = .51).

## Discussion

Opioid use has become a health care epidemic in the United States with increasing prescription rates in recent years, and more than 3% of adults reporting long-term opioid use.^16^, ^17^, ^18^ Patients taking opioids long term are more likely to endorse bowel-related issues. Despite the known GI side effects of opioids, including delayed gastric emptying, previous studies have reported that up to 46% of patients with gastroparesis regularly use opioids.^3^, ^4^, ^5^ Although opioid discontinuation and alternative strategies for symptom management should invariably be the first-line treatment, the reality is that most patients presumably fail to do so. In this study, we demonstrate that G-POEM may be considered for compassionate treatment for symptomatic patients with gastroparesis who are unable to discontinue long-term opioid use.

G-POEM has emerged as a potential therapy for patients with medically refractory gastroparesis, with recent randomized trials supporting its efficacy and safety.^19^^,^^20^ However, data on G-POEM clinical outcomes in patients on chronic opioids are limited. In our study, clinical response to G-POEM was achieved in 60.5% of the patients at a median follow-up of 6 months (range, 3-18 months), which is consistent with a recent systematic review reporting a 63.1% (95% confidence interval, 56.3%-69.5%) G-POEM response rate.^21^ Overall, these findings have important clinical implications. Current treatment options for patients with refractory gastroparesis on opioids are quite limited. Previous studies have demonstrated that other treatment options, including prokinetics and gastric electric stimulation, are less effective in patients with gastroparesis on opioids.^22^ We emphasize that the preferred approach to these patients should still be opioid cessation or reduction. Nonetheless, G-POEM may represent an adjunct therapy for some of these select patients, which by providing some symptom improvement, may help their efforts to reduce the use of opioids and lessen other narcotic-induced GI adverse effects. Additional studies, including long-term follow-up and post-treatment opioid use are underway and should help shed additional light on the role of G-POEM in this population.

Several studies have attempted to identify predictors of clinical and functional response to G-POEM among patients with gastroparesis. Among these, older age, shorter duration of symptoms, nondiabetic etiology, and symptoms other than abdominal pain as the cardinal manifestation, have been associated with treatment success.^22^ In our study, there was no significant difference in these variables between responders and nonresponders on chronic opioids. It remains to be determined whether prognostic variables may differ in this cohort of patients with refractory gastroparesis. Previous studies have suggested a potential role of FLIP and pyloric-directed therapies for gastroparesis. Desprez et al^23^ demonstrated benefit of intrapyloric botox in patients with DI <10.0 mm^2^/mm Hg at 40 mL. Similarly, Jacques et al^24^ identified a DI threshold of 9.2 mm^2^/mm Hg at 50 mL to be predictive of clinical response to G-POEM with 100% specificity and 72.2% sensitivity. In our study, we identified that patients who responded to G-POEM had a significantly lower preprocedure pyloric diameter and DI when compared with subjects who did not respond to the procedure. Notably, clinical response to G-POEM at follow-up was associated with a significant improvement in both pyloric diameter and DI, which was not seen among nonresponders. These findings are relevant because they further corroborate previous data suggesting an association between pyloric physiology as measured by FLIP and G-POEM outcomes. In a recent study by our group, we evaluated the histology of pyloric muscle biopsies in patients with gastroparesis undergoing G-POEM and identified that moderate to severe fibrosis was more prevalent among G-POEM nonresponders versus responders (81.3% vs 25%; *P* = .0002).^9^ It is plausible that patients with increased fibrosis tend to have lower pyloric compliance after pyloromyotomy and thereby lower clinical response to therapy. In all, our study adds to the growing body of evidence supporting FLIP as a potentially useful tool to assess pyloric sphincter characteristics before and after G-POEM and its association to clinical symptoms. Given that data on predictors of response to G-POEM remain variable, well-designed prospective studies are urgently needed to better elucidate reproducible markers that may improve patient selection.

We acknowledge the limitations of this study. First, we recognize that the overall sample size was relatively small; however, this is in direct relationship to the participating centers' treatment approach revolving around opioid cessation and only entertaining G-POEM as a compassionate treatment for gastroparesis in highly selected patients. Second, we acknowledge that clinical response was determined on the basis of the GCSI score, which has its limitations. Although the GCSI score has been commonly used as a standardized assessment tool for gastroparesis symptoms for research purposes, there is a lack of data to establish its utility as a surrogate for response to G-POEM in clinical practice. Importantly, the definition of clinical success varies slightly among the published data, limiting our ability to interpret and compare results across various studies. For our study, clinical response was determined on the basis of an improvement of ≥1 point in the average GCSI score, a widely accepted definition used in several other published studies.^13^^,^^14^ However, some studies have also used other variations. In all, future studies are needed to validate and standardize the use of the GCSI score as a parameter for clinical efficacy of G-POEM and how it may translate into a measurable and clinically relevant improvement. Third, this study lacked a placebo control group (ie, patients with gastroparesis not taking opioids). Nonetheless, our clinical response rate is consistent with those reported in various past studies, thereby providing external validity to our results. In addition, data collection on opioid use was determined by chart review and medication list. Chronic opioid use was defined as patients on daily opioids at the time of G-POEM. However, given the retrospective nature of the study, the accuracy of medication use (MME intake) before and after the G-POEM could not be corroborated outside what was documented in the electronic records. This is a significant limitation of the study because it remains unclear how these factors may have impacted study outcomes. Future studies designed to address the association between G-POEM and its impact on opioid requirements are needed. Similarly, data on other potential factors, including dietary modification after G-POEM and the concomitant use of other neuromodulator medications, were not captured as part of this study. This is another limitation as both variables could have impacted patients' symptoms. Lastly, we acknowledge that the approach to FLIP catheter insertion and placement varied between the 2 participating institutions, with 1 center using a through-the-scope approach and the other one advancing the catheter next to the endoscope with the assistance of a snare. With the more commonly used latter technique, we recommend advancing the catheter deep into the second portion of the duodenum. Once in the duodenum, inflation of the balloon should be initiated before complete withdrawal of the endoscope into the stomach. The inflating balloon provides some stability and reduces the risk of the catheter migrating into the stomach. Future studies are needed to help optimize and standardize FLIP measurements across the pylorus.

In conclusion, this study suggests that G-POEM is a safe and effective compassionate treatment for refractory gastroparesis among patients on chronic opioids. Although narcotic cessation and alternate pharmacologic or nonpharmacologic strategies should be the first-line intervention via a multidisciplinary approach, G-POEM may be an adjunct therapy, providing symptomatic benefit in select patients, particularly those with lower pyloric distensibility on physiologic testing. Future larger studies are needed to corroborate these initial findings and compare G-POEM with other treatment strategies for patients on chronic opioids and concomitant gastroparesis.

## Patient Consent

This article does not discuss individual patients, so no consent was needed.

## Disclosure

The following authors disclosed financial relationships: D. Yang is a consultant for 10.13039/100009734Olympus, 10.13039/501100002424Fujifilm, 10.13039/100008497Boston Scientific, 10.13039/100004374Medtronic, 3D-Matrix, Microtech, and Neptune Medical. D. Yang receives research grants from Microtech, 3D-Matrix, and 10.13039/100008497Boston Scientific. M.K. Hasan is a consultant for 10.13039/100008497Boston Scientific, 10.13039/100009734Olympus, and Microtech. N. Cosgrove is a consultant for 10.13039/100008497Boston Scientific and 10.13039/100009734Olympus. M.A. Arain is a consultant for 10.13039/100008497Boston Scientific, 10.13039/100009734Olympus, and 10.13039/100010479Cook Medical. J.H. Hwang is a consultant for 10.13039/100008497Boston Scientific, 10.13039/100009734Olympus, 10.13039/100004374Medtronic, Microtech, and Lumendi. All other authors disclosed no financial relationships.

## References

[1] Gonzaga E.R., Draganov P.V., Yang D. (2024). Gastric peroral endoscopic myotomy (G-POEM) for the management of gastroparesis. Tech Innov Gastrointest Endosc.

[2] Hirsch W., Nee J., Ballou S. (2019). Emergency department burden of gastroparesis in the United States, 2006 to 2013. J Clin Gastroenterol.

[3] Jehangir A., Parkman H.P. (2017). Chronic opioids in gastroparesis: relationship with gastrointestinal symptoms, healthcare utilization and employment. World J Gastroenterol.

[4] Hasler W.L., Wilson L.A., Nguyen L.A., Gastroparesis Clinical Research Consortium (2019). Opioid use and potency are associated with clinical features, quality of life, and use of resources in patients with gastroparesis. Clin Gastroenterol Hepatol.

[5] Siddiqui M.T., Bilal M., Schorr-Lesnick B. (2019). Opioid use disorder is associated with increased mortality and morbidity in patients with gastroparesis. Ann Gastroenterol.

[6] Biancuzzi H., Dal Mas F., Brescia V. (2022). Opioid misuse: a review of the main issues, challenges, and strategies. Int J Environ Res Public Health.

[7] Abell T.L., Camilleri M., Donohoe K. (2008). Consensus recommendations for gastric emptying scintigraphy: a joint report of the American American Neurogastroenterology and Motility Society and the Society of Nuclear Medicine. Am J Gastroenterol.

[8] Khan H.M., Brar T.S., Hasan M.K. (2023). Prospective study on the efficacy of endoscopic through-the-scope tack and suture system for gastric peroral endoscopic myotomy mucosal incision site closure. Endosc Int Open.

[9] Yang D., Hasan M.K., Bani Fawwaz B.A. (2024). Quantification of interstitial cells of Cajal and fibrosis during gastric per-oral endoscopic myotomy and its association with clinical outcomes. Endosc Int Open.

[10] Yang D., Kadkhodayan K., Arain M.A. (2022). Novel dual-action tissue through-the-scope clip for endoscopic closure. VideoGIE.

[11] Yim B., Gregor L., Siwiec R.M. (2023). Pyloric functional lumen imaging probe measurements are dependent on balloon position. J Neurogastroenterol Motil.

[12] Vosoughi K., Ichkhanian Y., Jacques J. (2020). Role of endoscopic functional luminal imaging probe in predicting the outcome of gastric peroral endoscopic pyloromyotomy (with video). Gastrointest Endosc.

[13] Gregor L., Wo J., DeWitt J. (2021). Gastric peroral endoscopic myotomy for the treatment of refractory gastroparesis: a prospective single-center experience with mid-term follow-up (with video). Gastrointest Endosc.

[14] Dacha S., Mekaroonkamol P., Li L. (2017). Outcomes and quality-of-life assessment after gastric per-oral endoscopic pyloromyotomy (with video). Gastrointest Endosc.

[15] Nass K.J., Zwager L.W., van der Vlugt M. (2022). Novel classification for adverse events in GI endoscopy: the AGREE classification. Gastrointest Endosc.

[16] Sheridan D.C., Laurie A., Hendrickson R.G. (2016). Association of overall opioid prescriptions on adolescent opioid abuse. J Emerg Med.

[17] Dunn K.M., Saunders K.W., Rutter C.M. (2010). Opioid prescriptions for chronic pain and overdose: a cohort study. Ann Intern Med.

[18] Wang Y.R., Fisher R.S., Parkman H.P. (2008). Gastroparesis-related hospitalizations in the United States: trends, characteristics, and outcomes, 1995-2004. Am J Gastroenterol.

[19] Martinek J., Hustak R., Mares J. (2022). Endoscopic pyloromyotomy for the treatment of severe and refractory gastroparesis: a pilot, randomized, sham-controlled trial. Gut.

[20] Gonzalez J.-M., Mion F., Pioche M. (2024). Gastric peroral endoscopic myotomy versus botulinum toxin injection for the treatment of refractory gastroparesis: results of a double-blind randomized controlled study. Endoscopy.

[21] Varghese C., Lim A., Daker C., BSM Consortium and GPOEM-GEMS Study Group (2024). Predictors of outcomes after gastric peroral endoscopic myotomy for refractory gastroparesis: a systematic review. Am J Gastroenterol.

[22] Maranki J.L., Lytes V., Meilahn J.E. (2007). Predictive factors for clinical improvement with enterra gastric electric stimulation treatment for refractory gastroparesis. Dig Dis Sci.

[23] Desprez C., Melchior C., Wuestenberghs F. (2019). Pyloric distensibility measurement predicts symptomatic response to intrapyloric botulinum toxin injection. Gastrointest Endosc.

[24] Jacques J., Pagnon L., Hure F. (2019). Peroral endoscopic pyloromyotomy is efficacious and safe for refractory gastroparesis: prospective trial with assessment of pyloric function. Endoscopy.

